# Supplementary material for: APOBEC3B-mediated corruption of the tumor cell immunopeptidome induces heteroclitic neoepitopes for cancer immunotherapy
Source: Nat Commun. 2020 Feb 7;11:790. doi: 10.1038/s41467-020-14568-7 (PMC7005822; doi:10.1038/s41467-020-14568-7)
Supplement: Supplementary file 2 — Supplementary Information [file 41467_2020_14568_MOESM2_ESM.pdf]

Supplementary Information

APOBEC3B-mediated Corruption of the Tumor Cell Immunopeptidome Induces Heteroclitic Neoepitopes for Cancer Immunotherapy

Driscoll et al.

Supplementary Figure 1

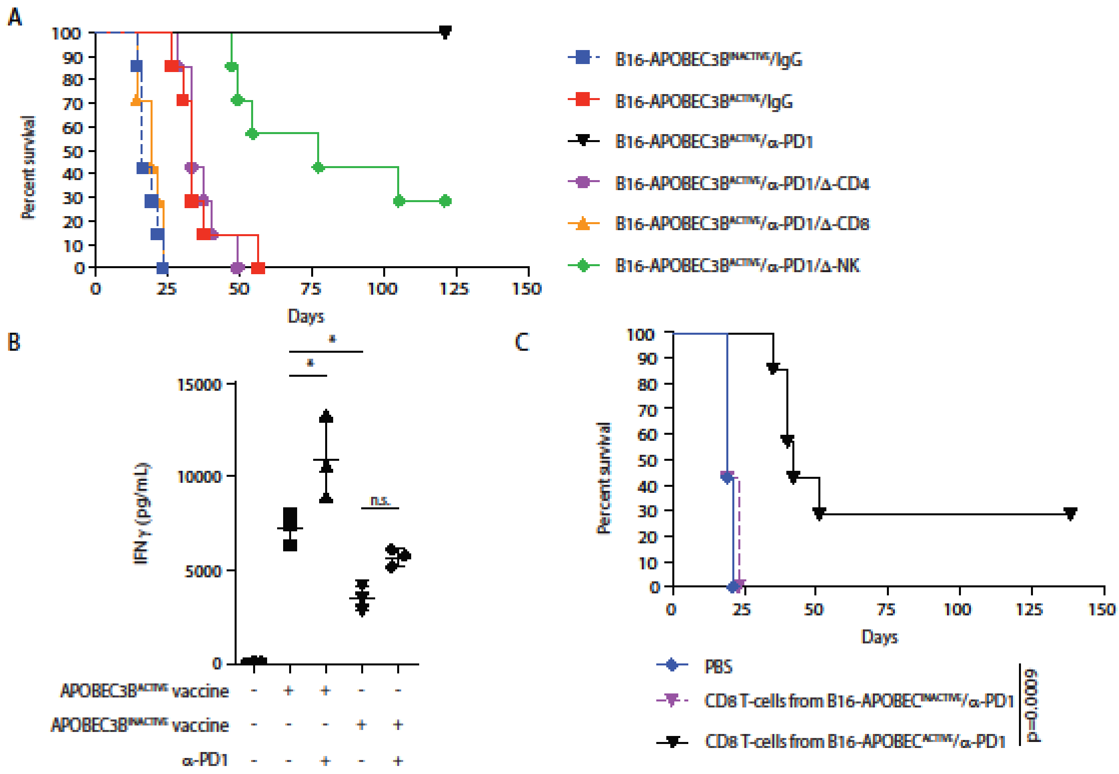

Supplementary Figure 1. CD4, CD8, and NK cells mediate APOBEC3B^{ACTIVE} vaccination.

(**A**) B16-APOBEC3B^{ACTIVE} vaccination and/or antibody-mediated checkpoint inhibition was performed as in Figure (**2A**), with the addition of antibodies depleting CD4 T cells, CD8 T cells, NK cells, or control IgG on day 4 after subcutaneous tumor implantation and weekly thereafter (n=7 mice/ group). (**B**) Spleens and lymph nodes obtained from mice (n=3 per group) succumbing to their disease in (**2B,C**) were made into single-cell suspensions and co-cultured with B16 target cells for 72 hours. Supernatant from the co-culture was assayed using a mouse interferon gamma ELISA. ANOVA used followed by Tukey's multiple comparison test. Error bars indicate mean and SD. * $p \leq 0.05$. (**C**) Spleens and lymph nodes obtained from mice treated with B16-APOBEC3B^{ACTIVE} vaccination and/or antibody-mediated checkpoint inhibition were made into single-cell suspensions and reinfused at dose of 1.2×10^7 cells intravenously per B16 subcutaneous tumor-bearing mouse (n=7 mice per group). Groups were compared using a Log-Rank test.

Supplementary Figure 2

A

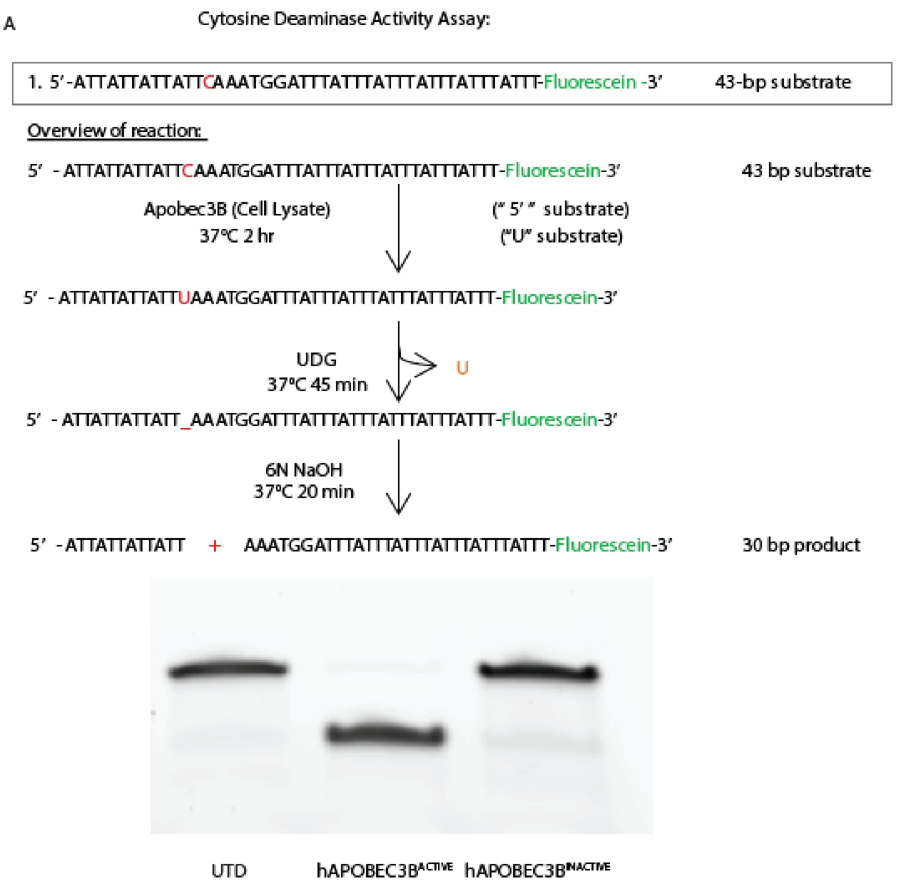

B

	C-T or G-A Transitions	Total Single Nucleotide Changes	Indels	Amino Acid Changes	Amino Acid Changes (APOBEC)
Number	68,447	861,417	419,722	3308	244
Percent Clonality	25.50	23.42	31.47	23.46	23.0

Supplementary Figure 2. Mutational activity of APOBEC3B^{ACTIVE} compared to APOBEC3B^{INACTIVE} cell transduction.

(A) Lysates of APOBEC3B^{ACTIVE} cells, but not APOBEC3B^{INACTIVE} cells, display APOBEC3B-charateristic deamination activity. Lysates of untransduced (UTD) parental, APOBEC3B^{ACTIVE} or APOBEC3B^{INACTIVE} cells were tested for their ability to deaminate a single C to a U residue within a 43bp fluorescein labeled probe, which, in the presence of 6N NaOH, would be cleaved into a 30bp (labeled) and 12bp (unlabeled) product (see **Methods**). **(B)** The different types of mutations found in APOBEC3B^{ACTIVE}, compared to APOBEC3B^{INACTIVE}, cells, along with the mean percentage clonality of all of such mutations in each group, is shown.

Supplementary Figure 3

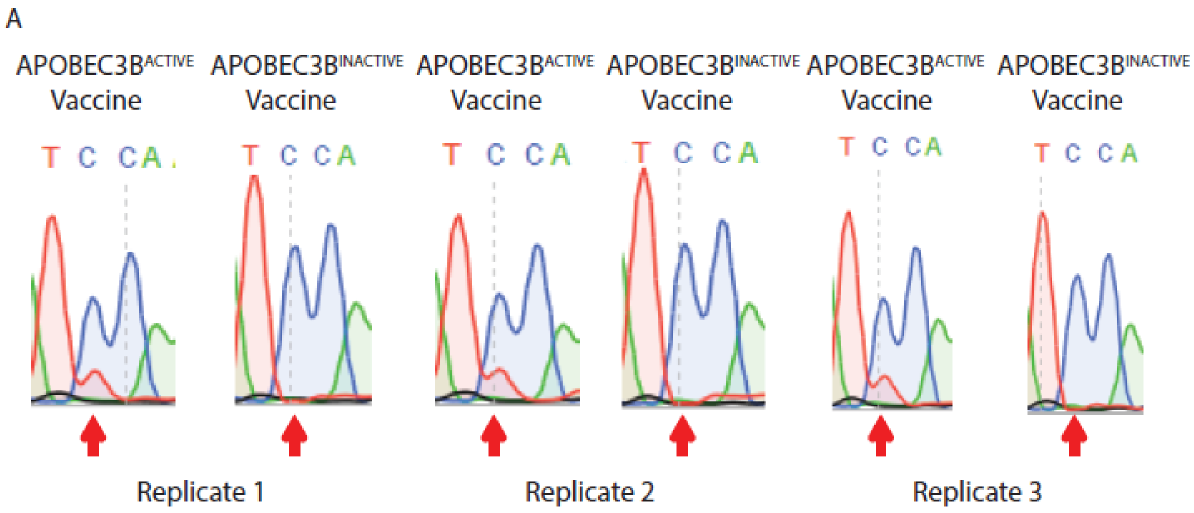

Supplementary Figure 3. Detection of CSDE1 mutation in vaccine preparations.

(**A**) Sanger sequencing of CSDE1 from B16-APOBEC3B^{ACTIVE} or B16-APOBEC3B^{INACTIVE} vaccine preparations was performed in three independent experiments. Each preparation of the B16-APOBEC3B^{ACTIVE} vaccine had a proportion of the cells containing a C with another containing a T at the thirteenth base pair, corresponding to the P5S amino acid change.

Supplementary Figure 4

A

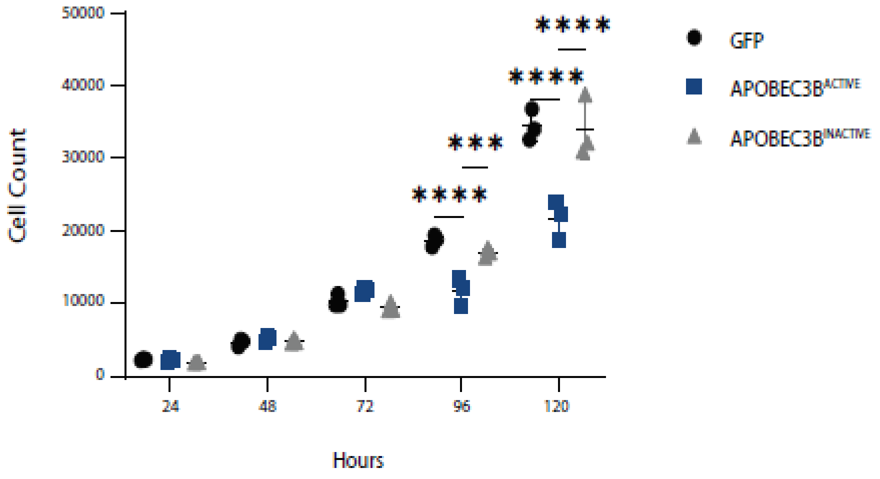

B

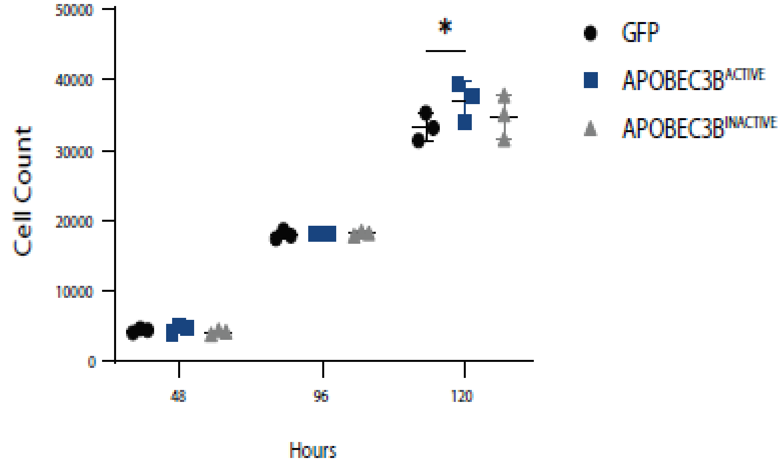

Supplementary Figure 4. Transient APOBEC3B overexpression is toxic to tumor cells.

(**A**) 10^4 B16tk cells were infected with retroviral vectors pBabe GFP; pBabeAPOBEC^{ACTIVE} or pBabeAPOBEC^{INACTIVE} at an MOI of ~10. 24hrs later, infected cells were harvested, washed and re-plated at 10^3 cells per well in triplicates. Cell growth was monitored over a period of 120hrs as shown. Error bars indicate mean and SD.*** $p \leq 0.001$ ****$p \leq 0.0001$. (**B**) At the 120hr timepoint, surviving cells were harvested and pooled from the triplicates of each treatment. 10^3 cells were re-plated in triplicates and cell growth measured over a further period of 120hrs as shown. Error bars indicate mean and SD. * $p \leq 0.05$.

Supplementary Figure 5. Gating scheme for figure 6B middle panel

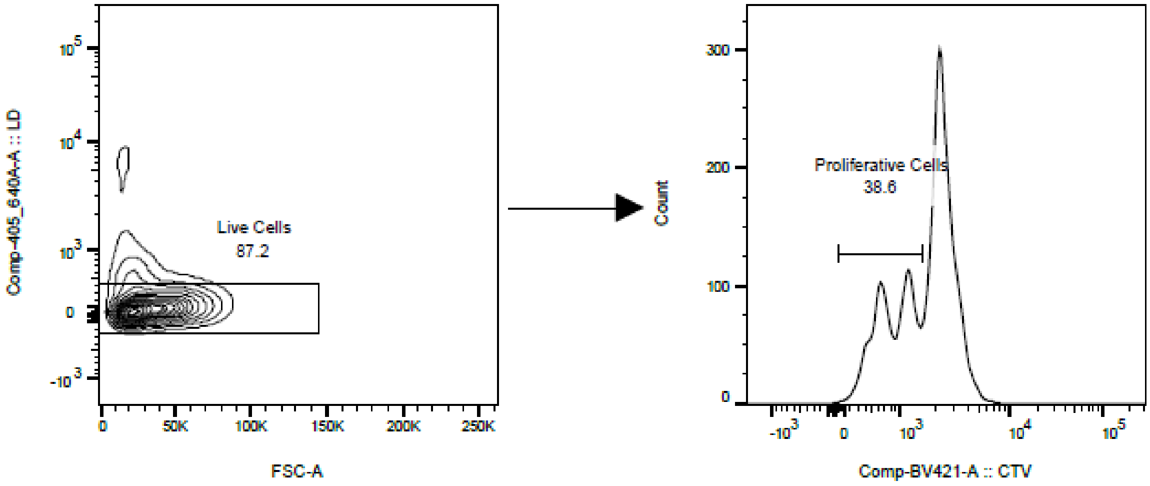

Supplementary Table 1

Gene	variant_no	HLA	peptide	pep_ID	Affinity(nM)	peptide_MT	Affinity(nM)_MT	differentialMTsubtractWT	logFCMT_WT
Ahctf1	14	H-2-Db	NSAANLRFV	WT_A578T_Ahctf1	615.66	NSATNLRFV	469.31	-146.35	0.762287626
C77080	31	H-2-Kb	ASFIFSKG	WT_A903T_C77080	602.55	TSFIFSKG	491.96	-110.59	0.816463364
Csde1	86	H-2-Kb	MSFDPNLL	WT_P5S_Csde1-00	587.81	MSFDSNLL	345.25	-242.56	0.587349654
Fcgbp	111	H-2-Kb	RSEELCPL	WT_L715F_Fcgbp-	4640.94	RSEEFCPL	129.99	-4510.95	0.028009412
Plbd2	299	H-2-Kb	SSGGWAARA	WT_A16V_Plbd2-0	4202.63	SSGGWAARV	440.6	-3762.03	0.104839113
Smc4	338	H-2-Db	SSVIDEISV	WT_D767N_Smc4-0	4439.13	SSVINEISV	56.05	-4383.08	0.012626348
Stag2	356	H-2-Kb	VRLKCLTAL	WT_L345F_Stag2-	1084.75	VRFKCLTAL	256.26	-828.49	0.236238765
Xpo1	404	H-2-Kb	TLVYLTHL	WT_L464F_Xpo1-2	969.17	TLVYFTHL	47.79	-921.38	0.049310235
Xpo1	404	H-2-Kb	VYLTHLDYV	WT_L464F_Xpo1-2	638.67	VYFTHLDYV	198.86	-439.81	0.311365807
Xpo1	404	H-2-Kb	RETLVYLTHL	WT_L464F_Xpo1-2	782.12	RETLVYFTHL	99.65	-682.47	0.127410116

Supplementary Table 1. MHC binding affinities for heteroclitic neoepitope candidates.

Binding affinities for the top ten heteroclitic neoepitope candidates with differential binding affinity to either H-2-Db or H-2-Kb and also expressed in the skin.
