# Supplementary material for: APOBEC3B-mediated corruption of the tumor cell immunopeptidome induces heteroclitic neoepitopes for cancer immunotherapy
Source: Nat Commun. 2020 Feb 7;11:790. doi: 10.1038/s41467-020-14568-7 (PMC7005822; doi:10.1038/s41467-020-14568-7)
Supplement: Supplementary file 3 — Description of Additional Supplementary Files [file 41467_2020_14568_MOESM3_ESM.pdf]

Description of Additional Supplementary Files

File Name: Supplementary Data 1

Description: Mutations from the whole genome sequencing of B16tk-APOBEC3BACTIVE-modified, VSVescaped population, compared to B16tk parental cells that resulted in a G to A or C to T transitions and an amino acid change.

File Name: Supplementary Data 2

Description: Missense mutations from Supplementary Data file 1 were translated into peptides with the missense mutation flanked by 10 amino acids on each side, as well as corresponding peptides with the wild type amino acid sequence.
